# Box-Behnken optimisation of growth performance, plasma metabolites and carcass traits as influenced by dietary energy, amino acid and starch to lipid ratios in broiler chickens

**DOI:** 10.1371/journal.pone.0213875

**Published:** 2019-03-21

**Authors:** Sonia Y. Liu, Victor D. Naranjo, Peter V. Chrystal, Johan Buyse, Peter H. Selle

**Affiliations:** 1 Poultry Research Foundation, Faculty of Science, The University of Sydney, Camden NSW, Australia; 2 School of Life and Environmental Sciences, Faculty of Science, The University of Sydney, Sydney NSW, Australia; 3 Evonik Nutrition and Care, Essen, Germany; 4 Baiada Poultry Pty Limited, Pendle Hill, NSW, Australia; 5 Laboratory of Livestock Physiology, Department of Biosystems, KU Leuven, Leuven, Belgium; University of Illinois, UNITED STATES

## Abstract

A Box-Behnken designed study was completed to predict growth performance, carcass characteristics and plasma hormone and metabolite levels as influenced by dietary energy, amino acid densities and starch to lipid ratios in male broiler chickens. The design comprised three dietary energy densities (11.25, 12.375 and 13.5 MJ/kg), three digestible lysine concentrations (9.2, 10.65 and 12.1 g/kg) and three starch to lipid ratios (4.5, 12.25 and 20.0) in broiler diets based on maize and soybean meal. Each of thirteen dietary treatments was offered to 10 replicates of 15 birds per replicate floor pen or a total of 1,950 Ross 308 male broiler chickens from 21 to 35 days post-hatch. Increasing dietary energy decreased feed intake with a quadratic relationship between feed intake and dietary standardised ileal digestible (SID) Lys concentrations, where increasing SID Lys initially increased and then depressed feed intake. Increasing dietary amino acid density increased body weight gain and carcass weight; however, dietary energy did not influence body weight gain, carcass and breast meat weight. Feed efficiency was positively influenced by energy and amino acid densities but negatively influenced by starch to lipid ratios and energy and amino acids had more pronounced impacts than starch to lipid ratios. This study indicated that both energy and amino acid densities regulate feed intakes in broiler chickens. Body weight gain of modern broiler chickens is more responsive to amino acid densities; nevertheless, dietary energy density continues to play an important role in protein utilisation, as reflected in significantly reduced plasma uric acid levels.

## Introduction

Satisfactory muscle protein deposition requires both glucose and amino acids because glucose is the primary energy source to promote the incorporation of amino acids into protein [1]. Liu and Selle [2] considered starch and protein digestive dynamics and proposed that glucose and amino acids should be made available in appropriately balanced quantities at sites of protein synthesis for efficient growth performance. Modern broiler chickens are selected for optimal growth rate, feed conversion efficiency and breast meat yield and are very responsive to both energy and amino acid dietary densities [3–5]. This emphasises the importance of energy and amino acid densities in the formulation of practical broiler chicken diets where these densities are usually considered in tandem. However, nutritionists may choose different approaches, which includes either keeping standardised ileal digestible (SID) lysine (Lys) constant or, alternatively, varying energy and amino acids individually in order to take better advantage of so-called ‘least-cost’ feed formulations. Additionally, the influence of proportions of the two major energy sources, including starch and lipid, gained very little attention in the literature. Our recent study [6] considered the former approach where broiler chickens were offered diets containing consistent digestible lysine to metabolisable energy ratios and the influence of starch to lipid ratios and dietary energy densities on growth performance was investigated. Other recent publications also investigated the influence of dietary protein to energy ratios on growth performance and efficiency of protein utilisation in chickens [5, 7]. However, there is a lack of recent studies investigating the interactive influence of energy and amino acids in broiler chickens when energy and amino acids are manipulated separately.

Moreover, Liu et al. [8] provided male broiler chickens with the choice of three diets containing 17.5 g/kg SID lysine and three levels of energy densities (11.04, 12.58 and 14.12 MJ/kg). All the other essential amino acids were balanced to constant ratios of SID Lys and the three diets were offered to broiler chickens simultaneously or sequentially (one at a time). From day 10 to 31 days post-hatch, simultaneously fed birds exhibited an 8.3% significantly lower feed conversion ratio (FCR) than those offered diets sequentially (1.217 versus 1.327, P < 0.0001) and consumed the high energy diet (14.12 MJ/kg) most in order to balance the high dietary amino acid density. This preliminary study led to our interest in exploring the impact of energy and amino acid separately on growth performance of broilers in order to enhance feed conversion efficiency and muscle protein deposition in broiler chickens to greater extents.

Energy density is usually the most expensive component of poultry diets and tremendous efforts have been made to describe and quantify energy more precisely via different systems [9, 10]. However, the influence of different energy sources, which are mainly starch and lipid in diets for mono-gastric animals, is seldom investigated. Starch is the most abundant macronutrient in typical broiler diets and lipid has been reported to influence feed intake [7] due to its impacts on pellet quality [11] and/or rate of gastric emptying [12]. Therefore, in comparison to our previous study [6], the present study evaluated the influence of dietary energy, amino acid densities and starch to lipid ratios on growth performance, carcass characteristics and plasma metabolites in broiler chickens during the grower phase. In order to study all the three factors at three different levels simultaneously, the multivariate optimization Box-Behnken response surface design was adopted in the present study.

## Materials and methods

### Experimental design

A 3-factor, 3-level Box-Behnken design (BBD) including 13 dietary treatments was used to determine the impact of energy (nitrogen-corrected apparent metabolisable energy, AMEn 11.25, 12.38, 13.50 MJ/kg), amino acid density (SID Lys, 9.20, 10.65, 12.10 g/kg) and starch to lipid ratio (S:L, 4.50, 12.25, 20.00 g/g) on growth and carcass parameters of male broilers from 21 to 35 days post-hatch. The independent variables and their low, medium, and high levels used in the Box-Behnken response surface design are shown in Table 1 and dietary treatments are listed in Table 2. The values of the centre points for each factor were 10.65 g/kg SID Lys, 12.38 MJ/kg AMEn and 12.25 S:L ratio as described for treatment 7. The evaluated responses (dependent variables) were parameters of growth performance and metabolites data in blood, including feed intake, weight gain, feed conversion ratio (FCR) and blood plasma concentrations of corticosterone, triacylglycerols, glucose, non-esterified fatty acids (NEFA), uric acid and insulin.

**Table 1:** Experimental factors and levels used in the Box-Behnken design.

Factors	Level (-)	Level (0)	Level (+)
*X*_*1*_: Digestible lysine concentration (g/kg)	9.2	10.65	12.1
*X*_*2*_: Energy density (AMEn MJ/kg)	11.25	12.375	13.5
*X*_*3*_: Starch: lipid ratios (g/g)	4.5	12.25	20.0

**Table 2:** The list of experimental treatments for broiler chickens from 21–35 days post-hatch.

Treatment	Code	Digestible lysine concentration (g/kg)	AMEn	Starch:lipid
			(MJ/kg)	(g/g)
1	− − 0	9.2	11.250	12.25
2	− 0 −	9.2	12.375	4.50
3	− 0 +	9.2	12.375	20.00
4	− + 0	9.2	13.500	12.25
5	0 − −	10.65	11.250	4.50
6	0 − +	10.65	11.250	20.00
7	0 0 0	10.65	12.375	12.25
8	0 + −	10.65	13.500	4.50
9	0 + +	10.65	13.500	20.00
10	+ - 0	12.1	11.250	12.25
11	+ 0 -	12.1	12.375	4.50
12	+ 0 +	12.1	12.375	20.00
13	+ + 0	12.1	13.500	12.25

#### Diet preparation

Experimental diets were based on maize, soyabean meal and wheat middlings with varying additions of maize gluten meal, soy protein concentrate and maize starch (Table 2). From 0 to 20 days post-hatch, all broilers were offered one common nutritionally adequate diet. Diets were formulated on SID basis and for each SID Lys level the ratios of other essential amino acid were balanced. Main ingredients were analysed for total amino acids, proximate chemical compositions in order to predict their AMEn by using WPSA equations [13]; then the estimated values were used for diet formulation. The formulation and calculated nutrient specifications of the experimental diets are shown in Tables 3 and 4. Experimental diets were pelleted and pellet durability index (PDI) was determined using the Holmen tester. Experimental diets did not contain prophylactic anti-microbial additives or enzymes in order to avoid confounding factors.

**Table 3:** Dietary formulations in experimental diets for broiler chickens from 21–35 days post-hatch.

Treatment (g/kg)	1	2	3	4	5	6	7	8	9	10	11	12	13
Maize	497	505	365	95	415	131	699	621	271	522	508	336	127
Soyabean meal, 48% CP	234	238	291	193	218	276	228	244	-	254	262	302	187
Wheat middlings	142	133	-	-	193	118	-	-	-	85	74	-	-
Maize starch	51	-	292	535	-	369	-	-	450	29	-	296	494
Maize gluten meal, 60% CP	-	-	-	50	-	-	-	-	73	-	-	-	50
Soy protein concentrate	-	-	-	50	-	-	-	-	130	-	-	-	50
Filler	30	30	3.9	-	80	50	11	21	-	50	50	5.2	-
Dicalcium phosphate 19	17	17	19	20	17	19	18	19	19	18	18	19	20
Limestome	8.1	8.0	6.8	6.8	8.3	7.0	7.8	7.6	8.1	7.8	7.7	6.7	6.9
Premix poultry	5.0	5.0	5.0	5.0	5.0	5.0	5.0	5.0	5.0	5.0	5.0	5.0	5.0
Soybean oil	5.0	52.5	5.0	28.7	43.5	5.0	8.9	63.7	9.9	5.0	51.8	5.0	26.8
Salt (NaCl)	2.9	3.0	3.4	2.4	2.2	2.7	2.3	2.4	1.0	1.9	2.0	2.4	1.1
Sodium bicarbonate	2.7	2.6	2.0	3.4	3.8	3.1	3.6	3.4	5.3	4.1	4.0	3.5	5.2
DL- Methionine	2.3	2.3	2.6	2.7	3.6	4.1	3.4	3.5	3.8	4.4	4.4	4.8	4.9
L-Lysine-HCl, 78%	1.3	1.2	0.7	2.0	3.6	3.0	3.6	3.3	6.0	4.6	4.5	4.1	5.9
L-Threonine	0.6	0.5	0.5	0.8	1.8	1.9	1.7	1.6	2.2	2.3	2.3	2.3	2.7
Potassium carbonate	0.4	0.4	2.0	3.8	0.4	1.6	2.1	2.1	6.0	0.7	0.7	1.8	3.8
Choline Cloride 60%	0.2	0.2	0.4	1.0	0.3	0.5	0.4	0.4	1.4	0.2	0.2	0.4	1.0
L-Valine	0.2	0.1	0.2	0.5	1.7	1.9	1.5	1.4	2.2	2.3	2.2	2.4	2.9
L-Isoleucine	-	-	-	-	1.4	1.3	1.2	1.1	1.8	1.8	1.8	1.7	2.1
L-Arginine	-	-	-	-	1.0	0.8	1.3	1.1	3.4	1.9	1.8	1.7	3.2
L- Tryptophen	-	-	-	-	-	-	-	-	0.5	-	-	-	0.3

**Table 4:** Calculated nutrient specifications in experimental diets for broiler chickens from 21–35 days post-hatch.

Treatment (g/kg)	1	2	3	4	5	6	7	8	9	10	11	12	13
AMEn, MJ/kg	11.25	12.38	12.38	13.50	11.25	11.25	12.38	13.50	13.50	11.25	12.38	12.38	13.50
Crude protein	180	180	180	180	180	180	180	180	180	195	195	195	195
Lipid	32.5	79.8	24.1	42.3	69.8	21.1	37	89.4	28.2	31.3	77.1	23.3	41.2
Crude fiber	32.2	31.7	19.5	12.1	34	23.8	24.3	23	11.8	28.5	27.5	19.2	12.5
Starch	398	359	482	518	314	422	453	402	563	383	347	467	505
Starch:lipid	12.2	4.5	20.0	12.2	4.5	20.0	12.2	4.5	20.0	12.2	4.5	20.0	12.2
Total Lys	10.2	10.2	10.1	10.1	11.6	11.6	11.5	11.5	11.4	13.1	13.1	13.1	13.0
Total Met	4.9	4.8	5.0	5.2	6.0	6.2	6.0	6.0	6.3	7.0	7.0	7.1	7.3
Total M+C	7.6	7.6	7.6	7.6	8.7	8.7	8.6	8.6	8.7	9.8	9.8	9.7	9.8
Total Thr	7.0	7.0	6.9	6.9	8.0	7.9	7.9	7.9	7.7	8.9	8.9	8.9	8.8
Total Trp	2.2	2.2	2.1	1.9	2.2	2.2	1.9	2.0	2.0	2.2	2.2	2.2	2.2
Total Arg	11.5	11.6	11.6	10.5	12.2	12.1	11.9	11.9	11.9	13.6	13.6	13.5	13.5
Total Ile	7.2	7.2	7.4	7.2	8.2	8.2	8.1	8.1	8.0	9.2	9.2	9.2	9.2
Total Val	8.4	8.4	8.3	8.3	9.5	9.5	9.4	9.4	9.3	10.7	10.7	10.6	10.6
SID Lys^1^	9.2	9.2	9.2	9.2	10.7	10.7	10.7	10.7	10.7	12.1	12.1	12.1	12.1
SID Met	4.6	4.6	4.8	4.9	5.8	6.0	5.8	5.8	6.1	6.8	6.8	6.9	7.1
SID M+C	6.9	6.9	6.9	6.9	8.0	8.0	8.0	8.0	8.0	9.1	9.1	9.1	9.1
SID Thr	6.0	6.0	6.0	6.0	6.9	6.9	6.9	6.9	6.9	7.9	7.9	7.9	7.9
SID Trp	1.9	1.9	1.9	1.7	1.9	1.9	1.7	1.7	1.7	1.9	1.9	1.9	1.9
SID Arg	10.5	10.6	10.8	9.7	11.2	11.2	11.2	11.2	11.2	12.7	12.7	12.7	12.7
SID Ile	6.4	6.5	6.7	6.4	7.5	7.5	7.5	7.5	7.5	8.5	8.5	8.5	8.5
SID Val	7.4	7.4	7.4	7.4	8.5	8.5	8.5	8.5	8.5	9.7	9.7	9.7	9.7
Ca	8.5	8.5	8.5	8.5	8.5	8.5	8.5	8.5	8.5	8.5	8.5	8.5	8.5
Avail. P	4.2	4.2	4.2	4.2	4.2	4.2	4.2	4.2	4.2	4.2	4.2	4.2	4.2
Na	2.0	2.0	2.0	2.0	2.0	2.0	2.0	2.0	2.0	2.0	2.0	2.0	2.0
Cl	2.5	2.5	2.5	2.5	2.5	2.5	2.5	2.5	2.5	2.5	2.5	2.5	2.5
K	7.7	7.7	7.7	7.7	7.7	7.7	7.7	7.7	7.7	7.7	7.7	7.7	7.7

^1^ standardised ideal digestible

### Bird management

All experimental procedures involving animals were approved by the Animal Ethics Committee of the University of Leuven (Trial number 15 53 16003). A total of 1,950 d-old commercial strain male Ross 308 broilers were obtained from a commercial hatchery and randomly assigned to 13 dietary treatments with 10 replicates of 15 birds per replicate pen. Five animal houses were used but complete randomised block design was conducted to include two replicates per dietary treatment or 26 pens per house. Fresh wood shavings were added to floor pens prior to the beginning of the feeding study. A common starter diet was offered to all the broiler chickens from 0 to 20 days post-hatch. At day 21, birds were allocated into floor pens on the basis of bodyweight to ensure the average initial body weights within each floor pen were nearly identical. Broiler chickens were offered the experimental diets from 21 to 35 days post-hatch. Birds were housed in an environmentally-controlled facility with unlimited access to feed and water. Lighting and environmental temperature programs strictly followed the 2014 Ross 308 bird management guidelines. Birds and feed were weighed again to determine weekly intake and FCR at 28 and 35 days post-hatch. Birds were checked daily and the incidence of dead or culled birds was recorded daily and their body-weights were used to adjust FCR calculations. FCR was calculated from feed intake divided by weight gain for the corresponding experimental period. Little score was evaluated at day 30 with the following five categories (score 0, dry and granular; score 1, dry and compact; score 2, wet and compact; score 3, completely wet and compact; score 3+, wet and sticky). All the assessment of growth performance and sample collection followed the order of pen number.

### Sample collection and chemical analysis

On 35 days post-hatch, 4 birds per pen were selected based on average pen weight for carcass measurements. Birds were individually weighed to obtain the coefficient of variation (CV) of each pen as the indicator of flock uniformity. Subsequently, birds were slaughtered and processed in a commercial processing plant and carcasses were weighed, breast muscle (*Pectoralis major*), wings and leg quarters were removed and weighed. Another 5 birds per pen were selected based on average pen weight. Blood was taken from a wing vein with a heparinized syringe and collected into iced tubes. After centrifugation, plasma was carefully removed and stored frozen (-70°C) until analysis for concentrations of corticosterone, triglycerides, glucose, NEFA, uric acid and insulin. The plasma corticosterone concentrations were measured using a Double Antibody Corticosterone ^125^ I RIA kit (MP Biomedicals, LLC, Orangeburg, NY 10962, USA). Plasma insulin concentrations were determined by using the Mercodia Mouse Insulin ELISA kit (Mercodia AB, SE-754 50 Uppsala, Zweden). Plasma metabolite concentrations were measured spectrophotometrically by using commercially available kits: glucose (LabAssay Glucose from Wako Pure Chemical Industries, Ltd., Osaka, Japan), triglycerides (Randox Triglycerides GPO-PAP Method, Randox Laboratories Ltd., Crumlin, United Kingdom), NEFA (NEFA-HR(2) ACS-ACOD Method, Wako Chemicals GmbH, 41468 Neuss, Duitsland), uric acid (Randox Uric Acid, Randox Laboratories Ltd., Crumlin, United Kingdom).

### Statistical analysis

The calculation of number of replicates was conducted by using Library *dae* in R 3.1.3 as described in Demetrio et al. [14]. The common variance of FCR according to our previous studies was expected to be 0.012. The number of replicates required to achieve a power of 0.8273, with a significance level of 0.05 in detecting a minimum difference of 0.16 in FCR is 10. Data were analyzed according to a 3-factor, 3-level BBD function using each pen of birds as the experimental unit. Response surfaces were fitted by first and second degree polynomial regressions in R 3.1.3. Akaike Information Criterion (AIC) was used for model comparison and selection. In the predicted model, the non-significant coefficients were excluded for recalculations of the reduced equations for each response variable. The primary experimental outcomes are growth performance including FCR and weight gain and the secondary experimental outcomes are plasma metabolites and carcass traits.

## Results

### Growth performance

The mortality rate during the experimental period was 0.87% which was not influenced by dietary treatment (P > 0.60). This mortality rate is extremely low comparing to industry practice and no adverse events were observed in the present study. The influence of dietary treatments on weight gain, feed intake and FCR from 21–35 days post-hatch is shown in Table 5 (S1 Table). The overall average FCR from 21–35 days post-hatch was superior to 2014 Ross 308 performance objectives by 1.8% (1.699 versus 1.731, P < 0.01) on the basis of one sample *t*-test. There was no significant influence on flock uniformity and litter score by changing starch to lipid ratios, energy and amino acid densities in broiler diets. Therefore, no coefficients and models were reported in Table 6 to predict flock uniformity (CV) and litter score. Table 6 shows the coefficient estimates and summary statistics of growth performance in response to dietary treatments. The response of feed intake (y) was described by the following equation,

$$y=58.063+533.94x_{1}-20.266x_{1}^{2}-10.008x_{1}x_{2}-0.5948x_{2}x_{3}$$

**Table 5:** Effects of dietary treatment on growth performance from 21–35 days post-hatch and flock uniformity and litter score at 35 days post-hatch.

Treatment	SID Lys	AMEn	S:L	Weight gain	Feed intake	FCR	CV	Litter score^1^
	(g/kg)	(MJ/kg)	(g/g)	g/bird	g/bird	g/g	(%)	(1–3)
1	9.2	11.25	12.25	1111	2133	1.928	8.9	2.2
2	9.2	12.375	4.5	1186	2084	1.761	7.3	2.1
3	9.2	12.375	20	1117	1979	1.777	7.0	2.1
4	9.2	13.5	12.25	1157	1905	1.648	7.9	2.2
5	10.65	11.25	4.5	1247	2200	1.766	7.8	2.2
6	10.65	11.25	20	1141	2143	1.879	7.8	2.0
7	10.65	12.375	12.25	1188	2056	1.736	7.3	2.1
8	10.65	13.5	4.5	1256	1972	1.571	7.2	2.0
9	10.65	13.5	20	1125	1812	1.612	7.8	2.2
10	12.1	11.25	12.25	1179	2062	1.750	8.5	2.1
11	12.1	12.375	4.5	1307	2049	1.568	6.6	2.3
12	12.1	12.375	20	1211	1923	1.588	6.7	2.3
13	12.1	13.5	12.25	1215	1817	1.497	7.3	2.1
			SEM	18.90	19.90	0.0213	0.60	0.13

^1^ score 1, dry and compact; score 2, wet and compact; score 3, moist and compact; score 3+, moist and sticky

**Table 6:** ANOVA, coefficient estimates and summary statistics of growth performance in response to digestible lysine concentration (SID Lys), energy density (AMEn) and starch to lipid ratios (S:L) in broiler chickens from 21–35 days post-hatch.

	Feed intake		Weight gain		FCR	
Variables^1^	Coefficient	P-Value	Coefficient	P-Value	Coefficient	P-Value
First order						
*X*_*1*_	533.94	1.55×10^−5^	29.371	4.72×10^−9^	0.26898	0.041
*X*_*2*_	-	-	-	-	-0.11074	< 2×10^−16^
*X*_*3*_	-	-	-18.209	1.02×10^−4^	0.01579	0.004
Second order						
*X*_*1*_	-20.266	3.88×10^−4^	-	-	-0.0155	0.0123
*X*_*2*_	-	-	-	-	-0.5198×10^−3^	0.016
*X*_*3*_	-	-	0.479	9.45×10^−3^	-	-
Interactions						
*X*_*1*:_ *X*_*2*_	-10.008	< 2×10^−16^	-	-	-	-
*X*_*2*:_ *X*_*3*_	-0.5948	1.13×10^−12^	-	-	-	-
*X*_*1*_:*X*_*3*_	-	-	-	-	-	-
Intercept	58.063	0.926	1008.5	< 2×10^−16^	1.887	0.008
R^2^	0.766		0.446		0.774	
R^2^_adj_	0.759		0.433		0.765	
P-value		<0.001		<0.001		<0.001

^1^*X*_*1*_: SID Lys (g/kg); *X*_*2*_: AMEn (MJ/kg); *X*_*3*_: S:L

**Fig 1:**
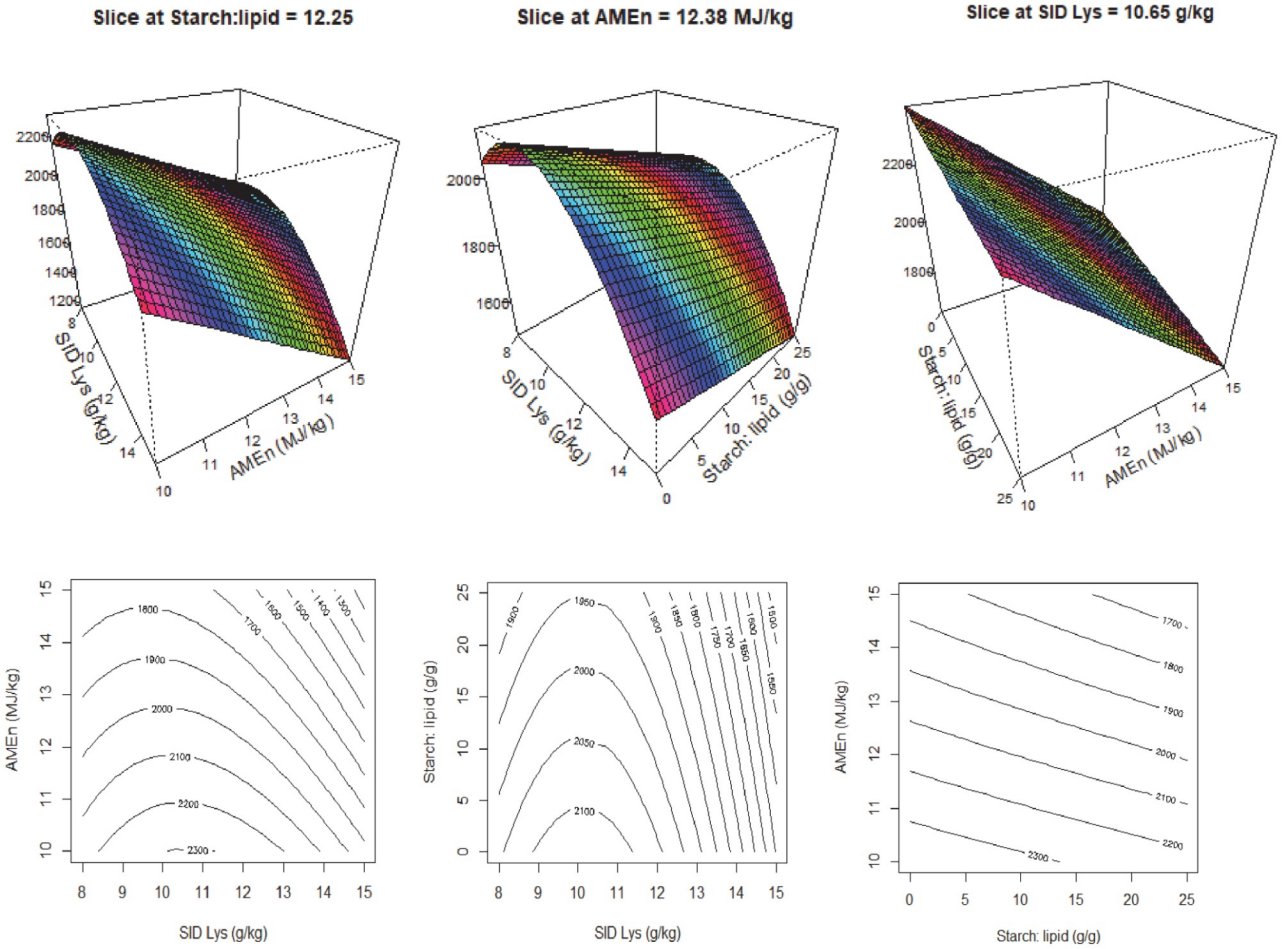
Response surface and contour plots describing relationships between feed intake (g/bird) and digestible lysine concentration (SID Lys), energy density (AMEn) and starch to lipid ratios (S:L) in broiler chickens from 21–35 days post-hatch.

Where *x*_1_ is the SID Lys concentration, *x*_2_ is the dietary AMEn (MJ/kg) and *x*_3_ is the S:L ratio. The response surface and contour plots for feed intake are illustrated in Fig 1. Increasing S:L ratios and dietary energy reduced feed intake; however, dietary energy density has more pronounced impact on feed intake than S:L ratios (Fig 1 right). There was a quadratic relationship between feed intake and dietary SID Lys concentrations, where increasing SID Lys first increased feed intake and then depressed feed intake (Fig 1 left and middle).

The response of weight gain (y) was described by the following equation,

$$y=1008.5+29.371x_{1}-18.209x_{3}+0.479x_{3}^{2}$$

**Fig 2:**
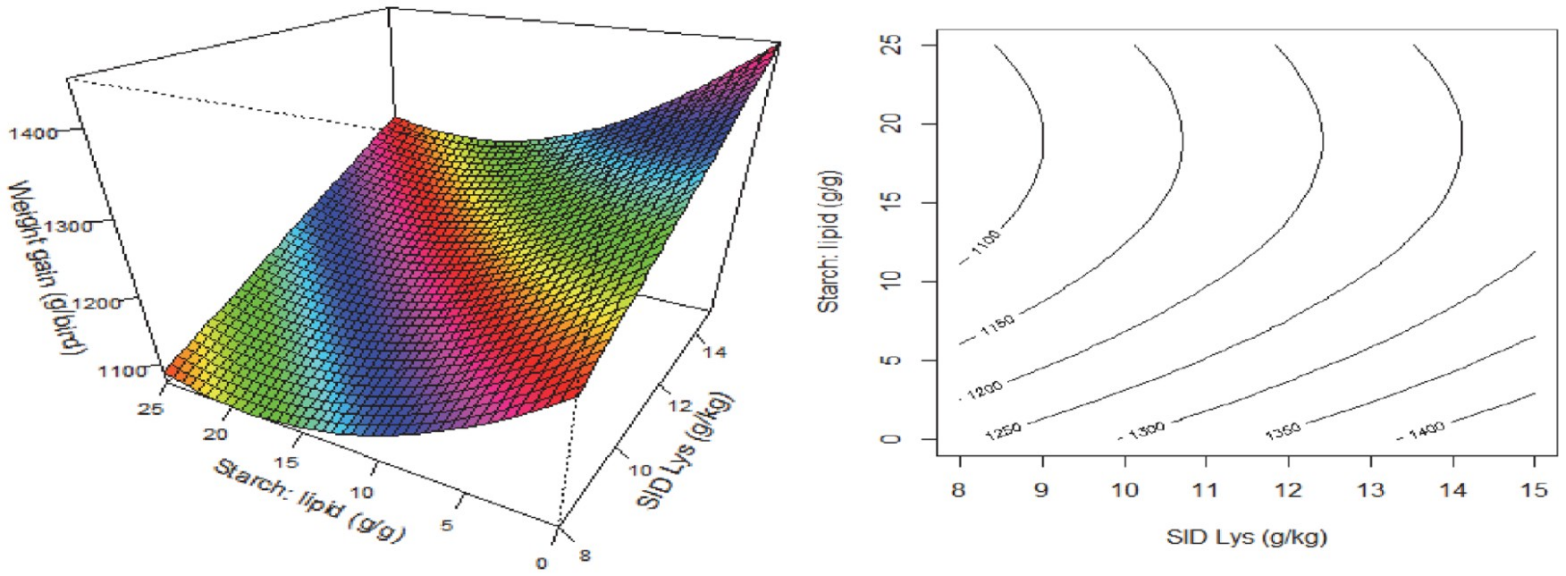
Response surface and contour plots describing relationships between weight gain (g/bird) and digestible lysine concentration (SID Lys) and starch to lipid ratios (S:L) in broiler chickens from 21–35 days post-hatch.

There were no interactions between the dietary variables nor dietary energy on weight gain from 21–35 days post-hatch. The response surface and contour plots for weight gain are illustrated in Fig 2. Increasing dietary SID Lys concentrations and decreasing S:L ratios increased weight gain in broiler chickens and dietary SID Lys concentrations had a more pronounced impact on weight gain than S:L ratios in broiler diets. Similar to feed intake, FCR was influenced by all the three dietary factors, and was predicted as,

$$y=1.887+0.26898x_{1}-0.11074x_{2}+0.01579x_{3}-0.0155x_{1}^{2}-0.5198\times 10^{-3}x_{2}^{2}$$

**Fig 3:**
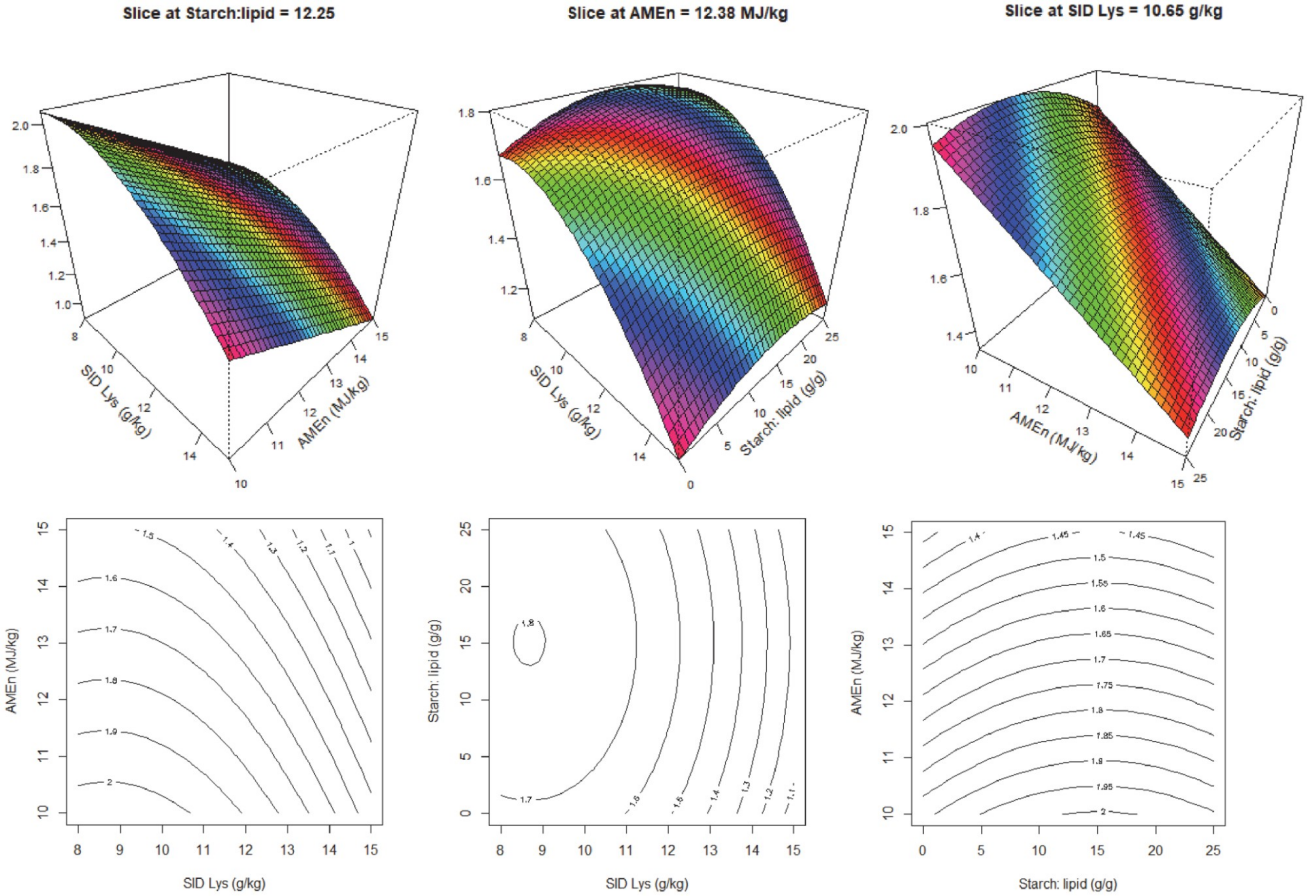
Response surface and contour plots describing relationships between FCR (g/g) and digestible lysine concentration (SID Lys), energy density (AMEn) and starch to lipid ratios (S:L) in broiler chickens from 21–35 days post-hatch.

There were no interactions between dietary S:L ratios, SID Lys and energy concentrations on weight gain or FCR. Increasing dietary energy or increasing SID Lys concentrations improved feed efficiency or reduced FCR (Fig 3 left). However, although increasing S:L ratios slightly increased FCR (Fig 3 middle), its impact on FCR was much less noticeable than dietary Lys and energy concentrations (Fig 3 middle and right).

### Carcass yields

The influence of dietary treatments on weights and yields of carcass, leg quarters, breasts and wings was tabulated in Table 7. The average live body weights (BW) following feed withdrawal was 2.272 kg/bird. The average weights of carcass, leg quarters, breasts and wings were 1357, 530, 476 and 133 g/bird, respectively; whereas, the average yields of carcass, leg quarters, breasts and wings were 64.5, 25.2, 22.6 and 6.3% of BW, respectively. Table 8 shows the coefficient estimates and summary statistics of carcass weights and yield data in response to dietary treatments. Carcass weight was only influenced by dietary Lys and S:L ratios and it was predicted as,

$$y=1069.3+29.3823x_{1}-2.0664x_{3}$$

**Table 7:** Effects of dietary treatment on slaughter live body weight (BW) and carcass traits in broiler chickens at 35 days post-hatch.

Treatment	SID Lys	AMEn	S:L	Live BW	Weight (g/bird)				Yield (% Live BW)			
	(g/kg)	(MJ/kg)	(g/g)	(kg/bird)	Carcass	Leg quarters	Breasts	Wings	Carcass	Leg quarters	Breasts	Wings
1	9.2	11.25	12.25	2.174	1315	506	455	136	64.2	24.7	22.2	6.6
2	9.2	12.375	4.5	2.228	1315	516	462	121	63.0	24.7	22.1	5.8
3	9.2	12.375	20	2.156	1292	508	439	133	64.1	25.2	21.8	6.6
4	9.2	13.5	12.25	2.211	1319	513	473	117	64.1	24.9	22.4	5.5
5	10.65	11.25	4.5	2.273	1384	544	477	142	64.6	25.4	22.2	6.6
6	10.65	11.25	20	2.203	1344	517	492	120	63.2	24.3	23.1	5.7
7	10.65	12.375	12.25	2.272	1377	542	479	145	64.5	25.4	22.4	6.8
8	10.65	13.5	4.5	2.272	1364	529	491	124	64.3	25.0	23.2	5.9
9	10.65	13.5	20	2.672	1347	538	449	143	64.8	25.9	21.6	6.9
10	12.1	11.25	12.25	2.207	1350	518	491	123	65.0	24.9	23.7	5.9
11	12.1	12.375	4.5	2.366	1441	568	498	146	65.2	25.7	22.5	6.6
12	12.1	12.375	20	2.243	1393	532	501	132	65.2	24.9	23.4	6.2
13	12.1	13.5	12.25	2.260	1399	554	480	145	65.9	26.1	22.6	6.8
			SEM	0.1287	11.0	5.3	5.8	1.6	0.67	0.34	0.30	0.17

**Table 8:** ANOVA, coefficient estimates and summary statistics of carcass traits (g/bird) in response to digestible lysine concentration (SID Lys), energy density (AMEn) and starch to lipid ratios (S:L) in broiler chickens.

	Carcass weight		Leg quarters		Breasts		Wings	
Variables^1^	Coefficient	P-Value	Coefficient	P-Value	Coefficient	P-Value	Coefficient	P-Value
First order								
*X*_*1*_	29.3823	< 2×10^−16^	-	-	-	-	3.4074	5.19×10^−10^
*X*_*2*_	-	-	-	-	-	-	-	-
*X*_*3*_	-2.0664	6.3×10^−5^	-7.4804	0.0995	-10.575	< 2×10^−16^	-	-
Second order								
*X*_*1*_	-	-	-2.6708	0.0010	-	-	-	-
*X*_*2*_	-	-	-2.9177	2.74×10^−6^	-	-	-	-
*X*_*3*_	-	-	-	-	-	-	-	-
Interactions								
*X*_*1*:_ *X*_*2*_	-	-	6.092	1.22×10^−5^	-	-	-	-
*X*_*2*:_ *X*_*3*_	-	-	-0.6048	0.0110	-	-	-	-
*X*_*1*_:*X*_*3*_	-	-	1.0467	0.0006	0.9224	6.96×10^−16^	-	-
Intercept	1069.3	< 2×10^−16^	493.97	< 2×10^−16^	485.1	< 2×10^−16^	96.6441	< 2×10^−16^
R^2^	0.2034		0.2103		0.1298		0.0723	
R^2^_adj_	0.2003		0.2010		0.1264		0.0705	
P-value		< 2×10^−16^		< 2×10^−16^		3.041*10^−16^		5.19×10^−10^

^1^*X*_*1*_: SID Lys (g/kg); *X*_*2*_: AMEn (MJ/kg); *X*_*3*_: S:L

**Fig 4:**
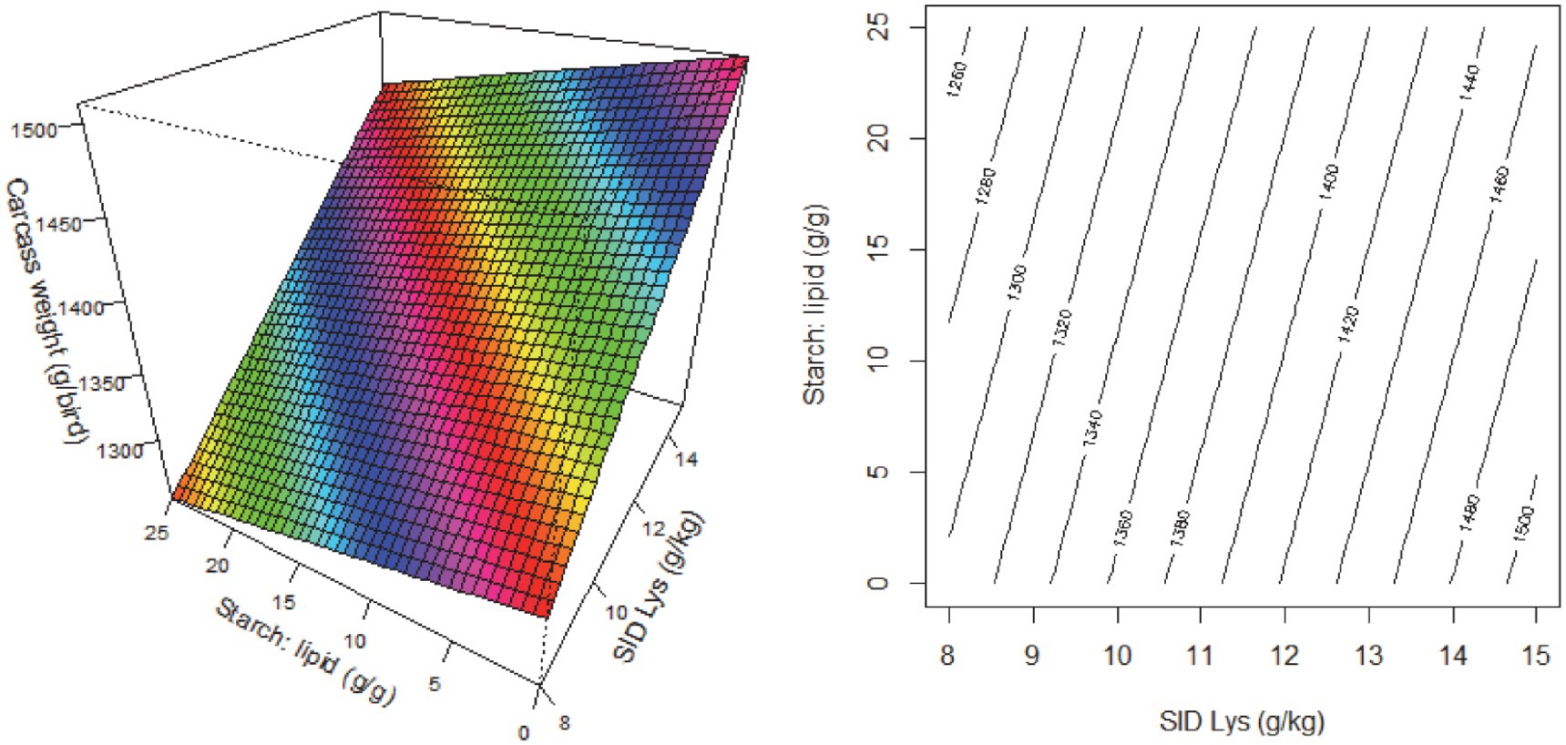
Response surface and contour plot describing relationships between carcass weight (g/bird) and digestible lysine concentration (SID Lys) and starch to lipid ratios (S:L) in broiler chickens.

As shown in Fig 4, dietary Lys had more impact on carcass weights than dietary S:L ratios and increasing SID Lys concentrations or decreasing S:L ratios increased carcass weights. Breast meat weights were not influenced by dietary energy density and it was predicted as,

$$y=485.1-10.575x_{3}+0.9224x_{1}x_{3}$$

**Fig 5:**
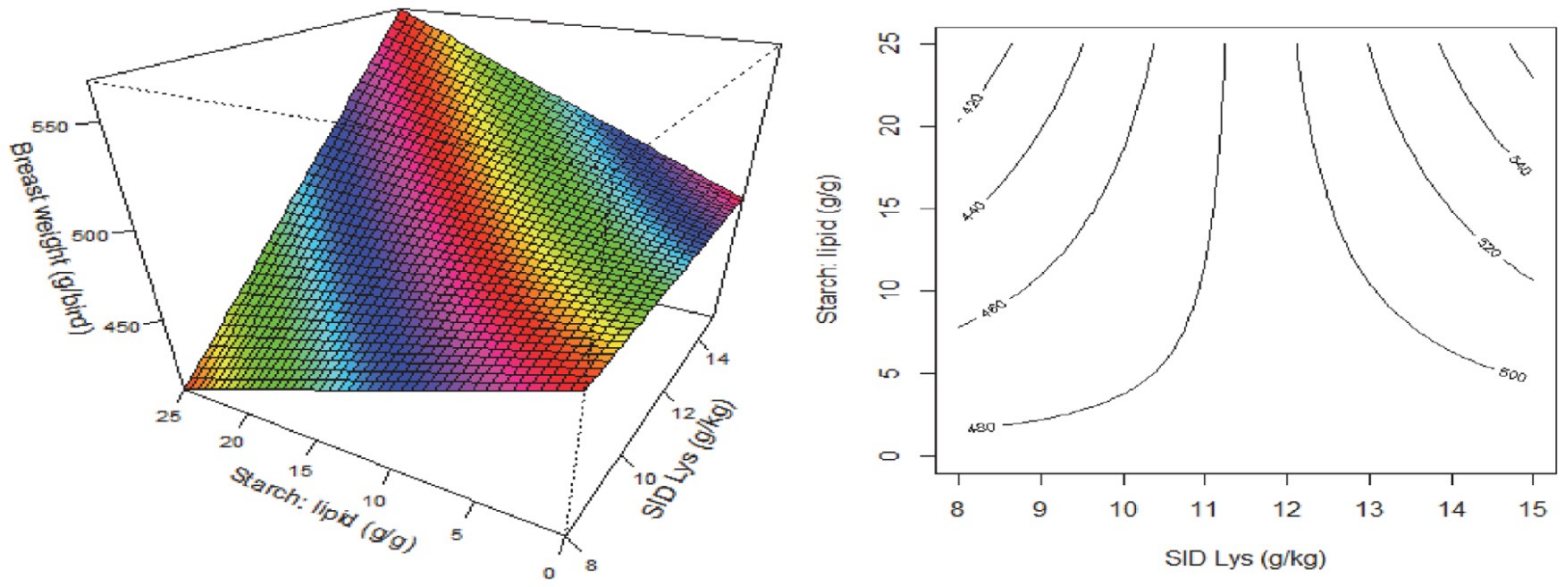
Response surface and contour plots describing relationships between breast weight (g/bird) and digestible lysine concentration (SID Lys) and starch to lipid ratios (S:L) in broiler chickens.

Fig 5 illustrates response surface and contour plots describing relationships between breast weight (g/bird) and SID Lys concentrations and S:L ratios in broiler chickens. There were interactions between dietary Lys and S:L ratios where the response to dietary amino acid densities depended on S:L ratios in the diet. When S:L ratio was low, increasing Lys concentrations slightly decreased breast meat weights, whereas when S:L ratio was high increasing Lys concentrations dramatically increased breast meat weights. The weights of leg quarters was predicted to be influenced by all the three dietary factors and the equation was,

$$y=493.97-7.4804x_{3}-2.6708x_{1}^{2}-2.9177x_{2}^{2}+6.092x_{1}x_{2}-0.6048x_{2}x_{3}+1.0467x_{1}x_{3}$$

**Fig 6:**
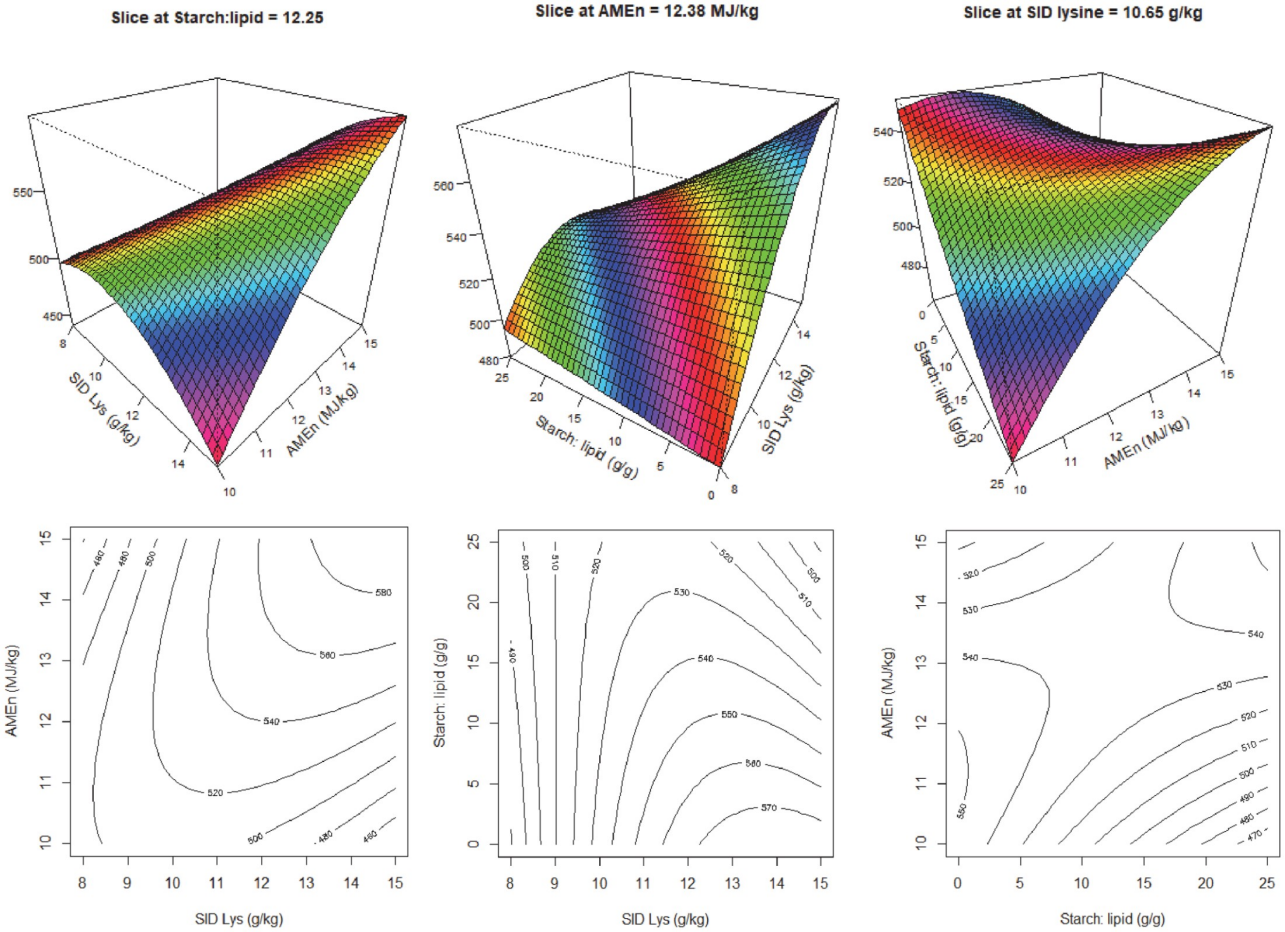
Response surface and contour plots describing relationships between leg quarters weight (g/bird) and digestible lysine concentration (SID Lys), energy density (AMEn) and starch to lipid ratios (S:L) in broiler chickens.

Fig 6 illustrates response surface and contour plots describing relationships between leg quarter weights (g/bird) and the three dietary variables in broiler chickens. There were interactions between the three dietary factors. Broiler chickens offered diets containing high energy and SID Lys concentrations were predicted to have higher leg quarter weights (Fig 6 left). Dietary S:L ratios had no obvious impact on leg quarter weights in diets containing low Lys concentrations; whereas in diets containing high Lys concentrations, decreasing S:L ratios was predicted to increase leg quarter weights (Fig 6 middle). In diets containing high S:L ratios, increasing dietary energy increased leg quarter weights; whereas in diets containing low S:L ratios, increasing dietary energy reduced leg quarter weights (Fig 6 right). The weights of wings were only influenced by dietary Lys concentrations where increasing SID Lys concentrations increased weights of wings. The prediction of the influence of dietary variables on carcass yields data as percentages of BW is reported in Table 9. There were only first order linear relationships between relative carcass yield and SID Lys. For example, carcass yield was positively correlated with dietary SID Lys concentrations (R^2^ = 0.2267, P = 0.0022). Relative leg quarters yield was positively correlated with dietary SID Lys and energy concentrations (R^2^ = 0.2165, P = 0.0124). Relative breast meat yield was positively correlated with dietary SID Lys concentrations (R^2^ = 0.2441, P = 0.0014). However, the relative yield of wings were not influenced by dietary treatments.

**Table 9:** ANOVA, coefficient estimates and summary statistics of carcass yields (% of live BW) in response to digestible lysine concentration (SID Lys), energy density (AMEn) and starch to lipid ratios (S:L) in broiler chickens.

	Carcass		Leg quarters		Breasts		Wings	
Variables^1^	Coefficient	P-Value	Coefficient	P-Value	Coefficient	P-Value	Coefficient	P-Value
First order								
*X*_*1*_	5.115	0.00218	1.8103	0.0482	3.1609	0.0014	-	-
*X*_*2*_	-	-	0.2741	0.0216	-	-	-	-
*X*_*3*_	-	-	-	-	-	-	-	-
Intercept	59.027	< 2×10^−16^	19.8393	8.73×10^−14^	19.19	< 2×10^−16^	-	-
R^2^	0.2267		0.2165		0.2441		-	-
P-value		0.0022		0.0124		0.0014	-	-

^1^*X*_*1*_: SID Lys (g/kg); *X*_*2*_: AMEn (MJ/kg); *X*_*3*_: S:L

### Blood metabolites

**Fig 7:**
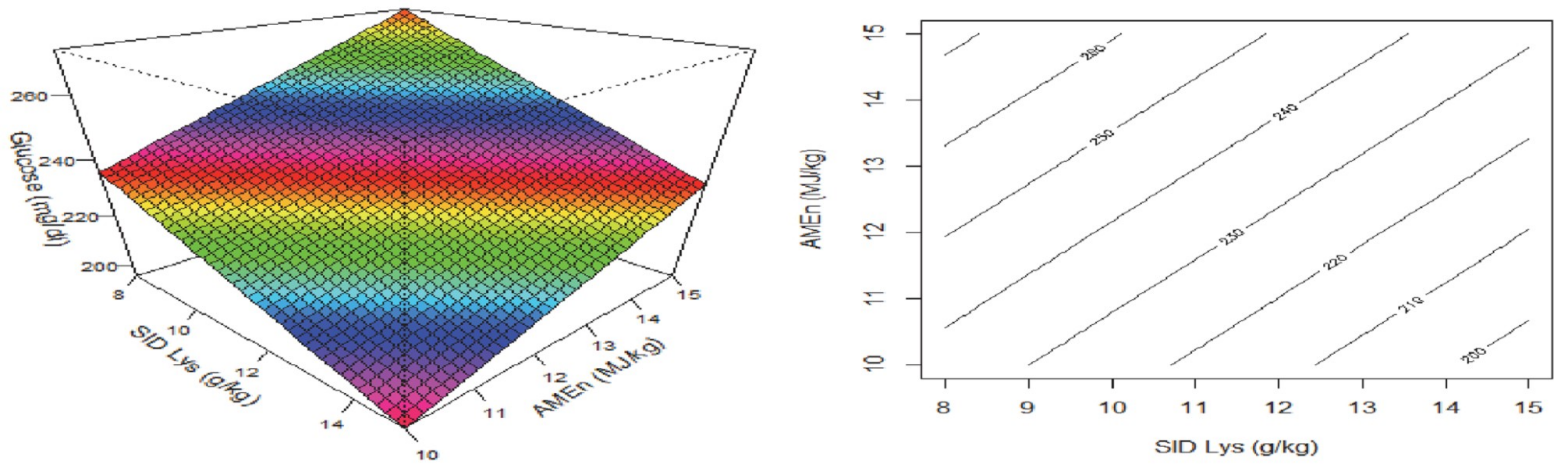
Response surface and contour describing relationships between blood glucose concentrations with dietary energy density and digestible lysine in broiler chickens.

The influence of dietary treatments on blood concentrations of corticosterone, triglycerides, glucose, NEFA, uric acid and insulin are shown in Table 10 and their relationships with dietary SID Lys, energy and S:L ratios are shown in Table 11. There was no dietary influence on concentrations of plasma corticosterone, triglycerides and insulin. Blood glucose concentration was negatively correlated (P = 0.007) with amino acid densities and positively correlated (P = 0.009) with energy density (Fig 7). NEFA concentrations in plasma was negatively correlated with S:L ratios in the diet (R^2^ = 0.076, P = 0.002) and uric acid in the plasma was negatively correlated with energy density where increasing dietary energy density decreased plasma uric acid concentrations (R^2^ = 0.076, P = 0.002).

**Table 10:** Effects of dietary treatment on blood parameters in broiler chickens at 35 days post-hatch.

Treatment	SID Lys	AMEn	S:L	Corticosterone	Triglycerides	Glucose	NEFA	Uric acid	Insulin
	(g/kg)	(MJ/kg)	(g/g)	(ng/ml)	(mg/dl)	(mg/dl)	(mM)	(mg/dl)	(ng/ml)
1	9.2	11.25	12.25	2.067	42.30	229.62	0.190	4.44	0.752
2	9.2	12.375	4.5	2.279	47.49	241.24	0.314	4.46	0.716
3	9.2	12.375	20	2.280	44.59	246.65	0.212	4.21	0.791
4	9.2	13.5	12.25	4.873	38.76	268.03	0.250	4.40	0.620
5	10.65	11.25	4.5	2.428	37.54	233.35	0.258	4.20	0.653
6	10.65	11.25	20	4.777	42.05	230.26	0.241	6.12	0.756
7	10.65	12.375	12.25	4.464	41.89	241.43	0.303	4.28	0.690
8	10.65	13.5	4.5	3.288	57.02	232.24	0.252	3.79	0.694
9	10.65	13.5	20	2.683	53.69	249.42	0.163	4.05	0.765
10	12.1	11.25	12.25	2.379	38.09	228.95	0.132	5.02	0.638
11	12.1	12.375	4.5	2.281	49.19	221.08	0.311	5.38	0.695
12	12.1	12.375	20	2.759	42.95	230.11	0.167	3.57	0.694
13	12.1	13.5	12.25	2.486	46.43	237.32	0.181	3.95	0.657
			SEM	0.7225	4.695	8.755	0.0323	0.409	0.1079

**Table 11:** ANOVA, coefficient estimates and summary statistics of blood parameters in response to digestible lysine concentration (SID Lys), energy density (AMEn) and starch to lipid ratios (S:L) in broiler chickens at 35 days post-hatch.

	Glucose		NEFA		Uric acid	
Variables^1^	Coefficient	P-Value	Coefficient	P-Value	Coefficient	P-Value
First order						
*X*_*1*_	-5.825	0.007	-	-	-	-
*X*_*2*_	7.294	0.009	-	-	-0.434	0.002
*X*_*3*_	-	-	-0.0051	0.002	-	-
Intercept	209.5	<0.001	0.291	<0.001	9.802	<0.001
R^2^	0.104		0.076		0.076	
R^2^_adj_	0.090		0.068		0.068	
P-value		<0.001		0.002		0.002

^1^*X*_*1*_: SID Lys (g/kg); *X*_*2*_: AMEn (MJ/kg); *X*_*3*_: S:L

## Discussion

**Fig 8:**
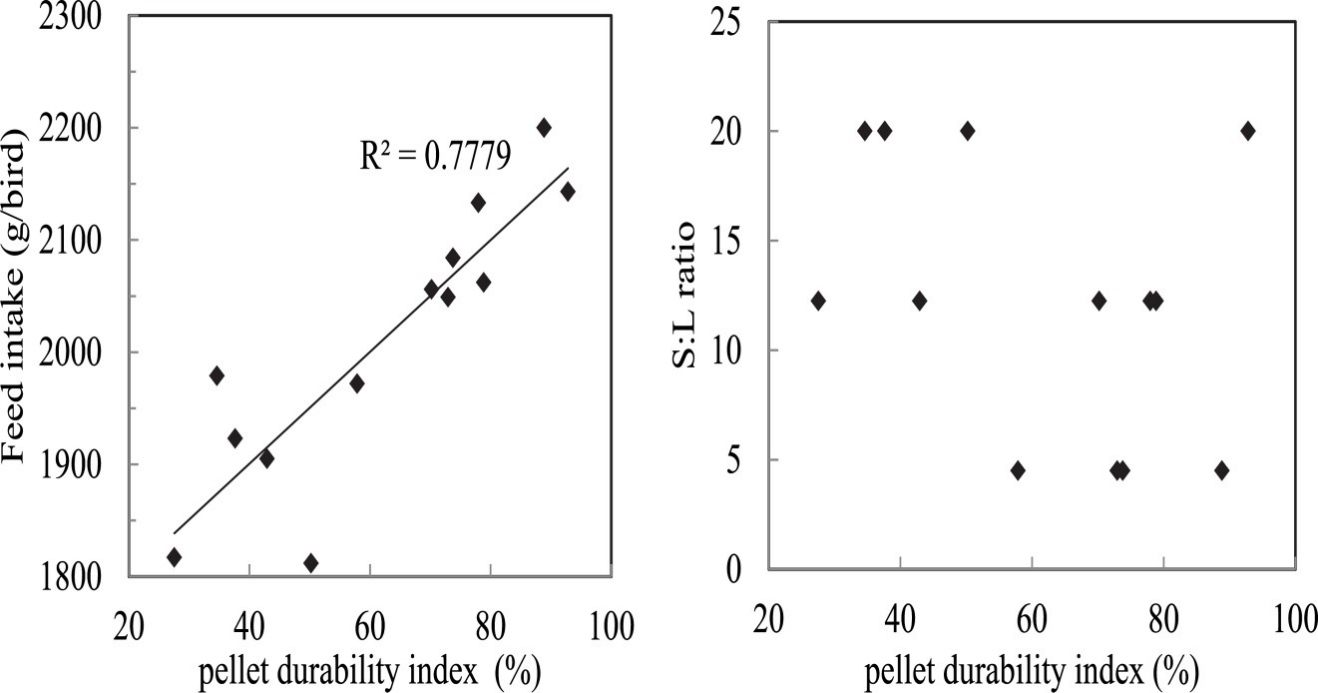
Linear regression between feed intake and pellet durability index (left) and the lack of correlation between dietary S:L ratios and pellet durability (right).

Before discussing the influence of dietary factors on feed intake, it is noteworthy that feed intake was positively correlated with pellet durability index (Fig 8 left, R^2^ = 0.78, P < 0.05) in the present study. However, there was no correlation between dietary S:L ratios and pellet durability index (Fig 8 right) which indicated the variations of dietary lipid content did not influence pellet durability. The importance of ‘pellet quality’ should not be overlooked as increasing pellet breaking force has been shown to improve weight gain and FCR in broiler chickens [15].

The conventional view is that the feed intake of broilers is regulated by the energy density of the diet [16], a flawed concept that is still held by many nutritionists. In the present study, feed intake was depressed by increasing energy and amino acids densities but there were interactions between dietary energy and amino acids densities, S:L ratios and energy densities (Fig 1). Over recent decades, the enhancement of productive traits in broiler chickens generated by genetic progress has been exponential [17]. Selection for increased growth rates, fuelled by increased feed intakes, has given birds the capacity to process increasing amount of nutrients. Classen [18] completed a series of studies where special attention was paid to confounding factors that usually overlooked in evaluations of the impact of dietary energy and amino acid levels on performance, including *in-vivo* energy measurements, consistent energy sources and pellet quality. This researcher concluded that energy levels did not influence feed intakes and that dietary energy levels need to be determined relative to the anticipated protein accretion of the bird [19]. Indeed, in the present study, feed intake was not influenced by energy density *per se* but there were interactions between dietary energy density and SID Lys concentration, dietary energy and S:L ratios (Table 6). In support of these findings, Gous et al. [5] reported a broken-stick response to protein to energy ratios in efficiency of protein utilisation and suggested animals often respond to the first limiting nutrient; hence, broilers exhibit an energy-dependent phase when high-protein feeds are offered or *vice versa*. Similarly, Gous [20] concluded birds are more likely to overconsume energy to meet other limiting nutrient(s) but their capacity to do so will depend on the degree to which nutrient/s are limiting and their ability to store the excess energy as body lipid and/or to increase heat production [21].

In the present study, increasing dietary S:L ratios slightly depressed feed intakes but their impact was less pronounced than dietary amino acid concentrations and, to a lesser extent, to the interaction between dietary energy and SID Lys and S:L (Fig 1). However, Khoddami et al. [6] reported that increasing dietary S:L ratios significantly increased feed intake. The possible explanation for this inconsistency may be differences in diet compositions and crude fibre concentrations. The formulated dietary fibre concentration ranged from 22.8 to 73.3 g/kg in the Khoddami et al. [6] study because diets were based on maize and oats, and crude fibre concentrations increased with decreasing dietary S:L ratios. However, the crude fibre concentration ranged only from 11.8 to 34 g/kg in the present study. Therefore, it is possible that broiler chickens offered diets containing high fibre content and low dietary S:L ratios had depressed feed intake as observed in Khoddami et al. [6] which was not the case in the present study. This may have been due to poor pellet quality resulting from higher crude fibre contents in the earlier study.

Despite the correlation between weight gain and feed intake (r = 0.275, P = 0.002), feed intake only explains about 7.5% of the variation in weight gain. Fig 2 shows that weight gain was influenced by dietary amino acid density and S:L ratios but not influenced by energy density and for a similar pattern exists for carcass yields (Fig 4). Increasing dietary S:L ratios decreased weight gain in broiler chickens in the present study which is not consistent with the Khoddami et al. [6]. However, as discussed, the difference may be due to the various impacts of dietary crude fiber and PDI on feed intake.

Feed conversion ratio was influenced by all three dietary factors where increasing energy and amino acid densities decreased FCR or enhanced feed efficiency. This is consistent with Khoddami et al. [6] where diets containing similar protein to energy ratios (0.87 g digestible lysine/MJ energy) but different nutrient densities (11.25, 12.38 and 13.50 MJ/kg) were offered to broiler chickens. Increasing dietary nutrient densities significantly reduced FCR from 1.533 to 1.298 in broilers from 7–27 days post-hatch. Similarly, in two earlier studies [7, 22], when iso-energetic diets were offered to broiler chickens, dietary protein had a pronounced impact on weight gain and FCR and increasing dietary protein concentrations reduced FCR in broiler chickens. However, in practice, the cost of feeding high density diets needs to be considered and balanced in order to assure profitability. In addition, it is important to note that high density diets may generate excessive metabolic stresses resulting in high mortality rates in broiler chickens. Liu et al. [8] reported a significantly higher mortality rate in simultaneously fed birds in comparison to sequentially fed birds (11% versus 0%) as the simultaneously fed birds consumed diets containing high energy and high amino acid densities to greater extents. As shown in Fig 3, the impact of dietary S:L ratios on FCR was relatively small in comparison to energy and amino acid densities which agrees with previous finding in Khoddami et al. [6] where dietary S:L did not influence FCR (P = 0.794) in broiler chickens from 7–27 days post-hatch.

Carcass weights were only influenced by dietary amino acid densities and S:L ratios, which was similar to body weight gain responses. Both increasing amino acid densities and decreasing S:L ratios enhanced carcass weights, but the impact of amino acid densities was more pronounced than that of dietary S:L ratios. Modern broiler chickens are genetically selected for growth rate and lean body mass gain, which demands routine updates of the amino acid requirements [23]. A number of studies have shown that broiler chickens are very responsive to variations in amino acid concentrations [3, 24, 25]. Reimer et al. [26] compared sexual maturation status at 21 weeks in broiler breeder pullets and reported that fat pad, as a percentage of total body weight, was 2.9, 4.5, 4.9 and 2.0% in the 2015, 1997, 1978 and 1957 strains, respectively (P < 0.001). This concluded that broiler chickens are genetically selected for lean body mass gain and the more recent strain has less body fat and higher lean body mass. Liu et al. [7] measured carcass composition by drying and grinding the whole carcass and found that the total fat carcass concentration was largely determined by the total protein intake, rather than the sum of starch and lipid intakes. Therefore, the higher proportion of lean body tissue in the more recent broiler strains may explain why body weight gain and carcass weight were influenced by amino acid densities but not energy. This is supported by Fig 5 which illustrates that breast meat weight was only influenced by amino acid densities and dietary S:L ratios.

In the present study, dietary treatment did not influence plasma triglyceride concentrations, which is considered to be an indicator of the hepatic *de novo* lipogenesis and fat deposition. Swennen et al. [27] found in broiler chickens offered low-protein diets that feed intake was increased to meet the protein requirements, leading to an involuntary overconsumption of energy (‘luxus’ energy consumption) compared to protein. Elevated plasma triglyceride concentrations are often observed because animals dealt with this excess energy intake by increasing *de novo* lipogenesis and fat deposition. Plasma uric acid concentrations are thought to indicate efficiency of protein utilisation (e.g. due to less protein/amino acid oxidation) and, in the present study, increasing dietary energy densities reduced plasma uric acid concentrations (P = 0.002), suggesting better protein utilisation in these broiler chickens. This observation supports the hypothesis that whilst dietary energy did not contribute directly to body weight gain (Fig 2) and breast meat weight (Fig 5), it is still pivotal for efficient protein utilisation. Increasing dietary energy densities with a concomitant increase in SID Lys and increase in S:L decreased feed intake and enhanced protein utilisation, thus improved feed efficiency as illustrated in Fig 3. The level of NEFA in the circulation is the net result of lipolysis on the one hand and, on the other, cellular uptake of NEFA for energy yield [28]. In the present study, increasing dietary S:L ratio decreased plasma NEFA concentrations (P = 0.002). Diets with high S:L ratios contained less lipid and may have led to lower lipid intake; therefore, the reduction of the net result between cellular fatty acid uptake and lipolysis was observed. Plasma glucose was reduced by dietary amino acid densities (P = 0.007) and increased dietary energy (P = 0.009) despite no direct influence of S:L ratios on glucose concentration (Fig 7). Swennen et al. [29] reported that when iso-energetic diets were offered, carbohydrate is the preferred energy source in comparison to lipid in broiler chickens offered low protein diets. However, dietary energy levels varied in the present study and this may have masked the influence of dietary S:L ratios on plasma glucose concentration. Response surface BBD and the interactive influence of protein, energy and sources of energy may have implications in other monogastric livestock animals; however, because of the physiological differences between human and poultry, outcomes from the present study may have limited relevance to human despite the importance of energy and amino acid nutrition in human.

## Conclusions

The interactions between dietary energy densities, amino acid and the energy source in respect of feed intake indicate that regulation of feed intake involves all three factors. This is different from the conventional view that energy is the sole influential factor for feed intake. In the present study, weight gain, carcass weight and breast meat were found to be influenced by amino acid concentrations but not energy density. This suggests that modern broiler chickens are more responsive to amino acid densities due to their increased lean body tissue composition as a consequence of selective breeding. However, plasma uric acid concentrations were reduced by increasing the energy concentration in the diet which indicated energy plays an important role in enhancing the efficiency of protein utilisation.

## Supporting information

S1 Table Raw data. (XLS)
